# Endovascular treatment of traumatic penile arteriospongious fistula in a patient with erectile dysfunction: a case report and literature review

**DOI:** 10.1186/s12894-023-01305-7

**Published:** 2023-08-09

**Authors:** Polona Vihtelič, Simon Hawlina, Peter Popovič

**Affiliations:** 1 Clinical Institute of Radiology, University Medical Centre Ljubljana, Zaloška cesta 7, Ljubljana, SI-1000 Slovenia; 2 Faculty of Medicine, University of Ljubljana, Vrazov trg 2, Ljubljana, SI-1000 Slovenia; 3 Department of Urology, University Medical Centre Ljubljana, Zaloška cesta 7, Ljubljana, SI-1000 Slovenia

**Keywords:** Arteriovenous fistula, Arteriospongious fistula, Erectile dysfunction, Endovascular embolization, Perineal trauma

## Abstract

**Background:**

An arteriovenous fistula is an abnormal communication between an artery and a vein. Traumatic penile arteriospongious fistula is a rare complication and has been described as a cause of erectile dysfunction. Clinical evaluation of patients with erectile dysfunction after penile trauma includes a thorough history, physical examination, vascular assessment, and other complementary exams. Treatment consists of endovascular embolization, surgical ligation, or a combination of both techniques.

**Case presentation:**

A 40-year-old man presented with erectile dysfunction that had persisted since suffering blunt trauma a few months ago. He reported problems with short duration of erection and insufficient penile tumescence. Due to high suspicion of an arteriovenous fistula, he was referred to angiography, which confirmed the diagnosis of an abnormal connection between the pudendal vessels. The patient was treated with the coil embolization technique and the symptoms were successfully resolved after endovascular treatment.

**Conclusions:**

The appearance of a post-traumatic arteriospongious fistula is a rare complication with almost non-existent literature reported. Rapid development in endovascular techniques, in which we use embolic agents to block anomalous blood flow, has allowed safe, effective and less invasive alternative to surgery. Our case demonstrates that endovascular approach is a successful treatment for post-traumatic arteriospongious fistula since the symptoms were resolved, and normal erectile function was regained after the intervention.

## Background

Erectile dysfunction is defined as a persistent inability to attain and maintain an erection for satisfactory sexual performance. The International Society of Impotence Research broadly classifies male erectile dysfunction into psychogenic and organic dysfunctions, which can be subdivided into vasculogenic (arteriogenic, cavernosal, mixed), neurogenic, anatomic, and endocrinologic erectile dysfunction [1]. Vascular injury is the most common etiology of organic erectile dysfunction [2].

An arteriovenous fistula is an abnormal communication between an artery and a vein. The anomalous passageway creates a low-resistance and high-flow system in which the blood flows directly from an artery into a vein, bypassing capillaries and therefore diminishing tissue perfusion [3]. Post-traumatic penile arteriovenous fistula manifests as high-flow priapism due to the formation of an arteriocavernosal fistula. Increased arterial blood flow input with normal venous return causes non-ischemic painless priapism [4, 5]. However, an arteriovenous shunt can also occur directly to the spongy body, resulting in an arteriospongious fistula. The spongy body is supplied by the bulbar artery, which is not directly linked to the process of erection but to penile tumescence. As a result of blood shunt, input arterial blood flow equalizes with output venous flow, resulting in pain and erectile deficit [4].

Treatment of traumatic arteriovenous fistula includes endovascular embolization or surgical ligation. Endovascular embolization with coils is a safe and effective treatment method since it allows accurate and safe closure of the aberrant communication between the artery and vein [6]. We present a case of a patient with a traumatic arteriospongious fistula causing erectile dysfunction and its successful endovascular occlusion with microcoils, which is not well documented in prior literature.

### Case presentation

A 40-year-old man presented to a urologic specialist with a primary concern of erectile dysfunction resulting from perineal blunt trauma during a bicycle accident three months earlier. The trauma occurred when the patient attempted to evade a collision, leading to a direct impact on his perineum by the bicycle’s frame. Subsequently, the patient reported a progressive decline in the duration of his erections and insufficient penile tumescence as persistent symptoms associated with the perineal injury. He underwent urologic evaluation, but clinical examination revealed no visible signs of penile injury. Based on the mechanism of injury and the gradual deterioration of erectile function, a high clinical suspicion of post-traumatic vascular damage prompted the decision to forgo penile Doppler ultrasound during the initial evaluation. Instead, the patient was referred directly for angiography, which provided a more detailed visualization of the vessels involved in penile erection, aiding in the assessment of potential vascular abnormalities.

**Fig. 1:**
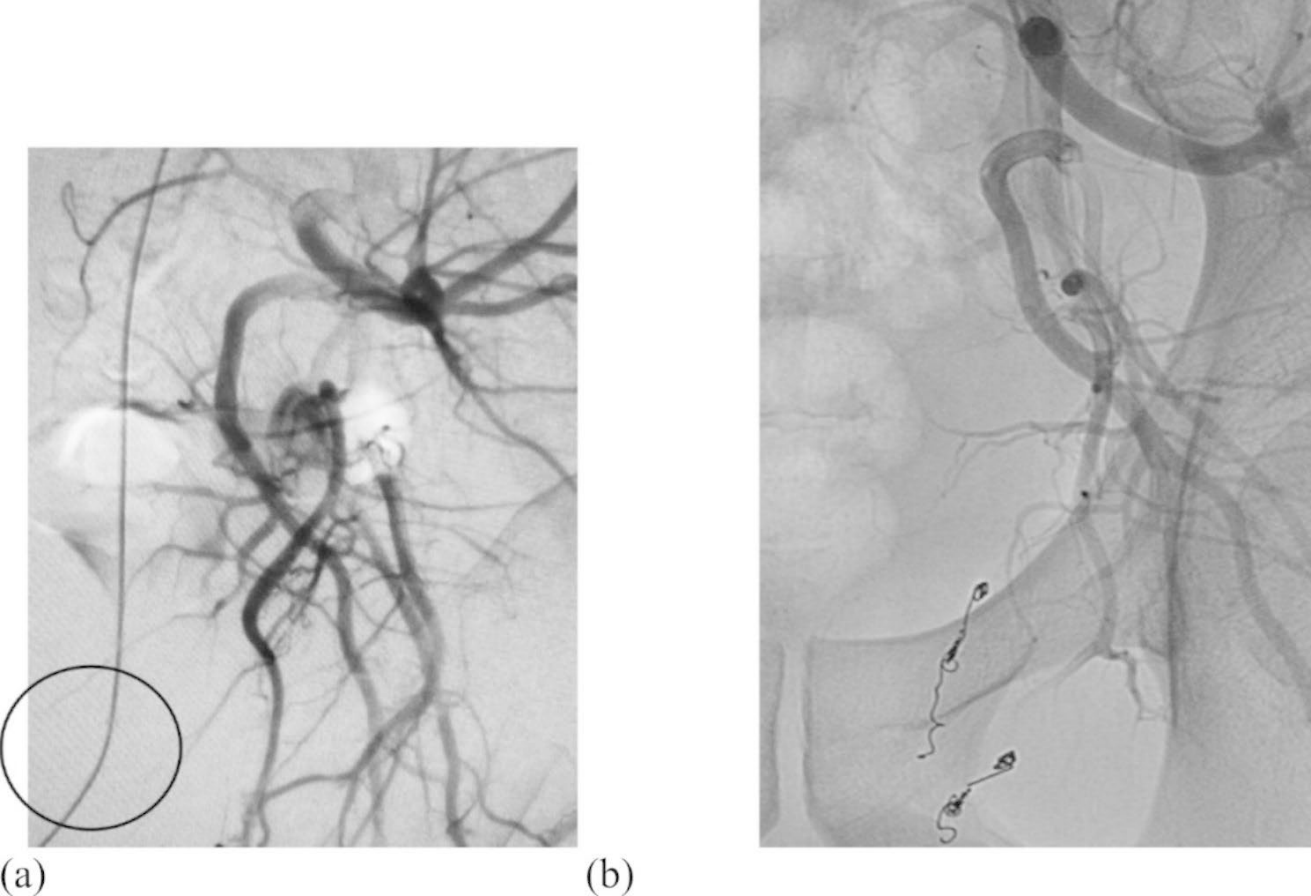
(**a**) Selective left iliac angiogram showing an arteriospongious fistula supplied by two branches of the pudendal artery. (**b**) Control angiogram after the first endovascular procedure demonstrating the position of microcoils

Digital subtraction angiography of the left internal iliac artery confirmed the presence of an arteriospongious fistula supplied by two branches of the pudendal artery (Fig. 1a). We performed superselective catheterization of the branches using a microcatheter (Progreat, Terumo, Belgium) and embolization with microcoils (Tornado, COOK) to exclude a fistula from systemic circulation. Post-embolization angiogram revealed that an aberrant communication was occluded (Fig. 1b).

**Fig. 2:**
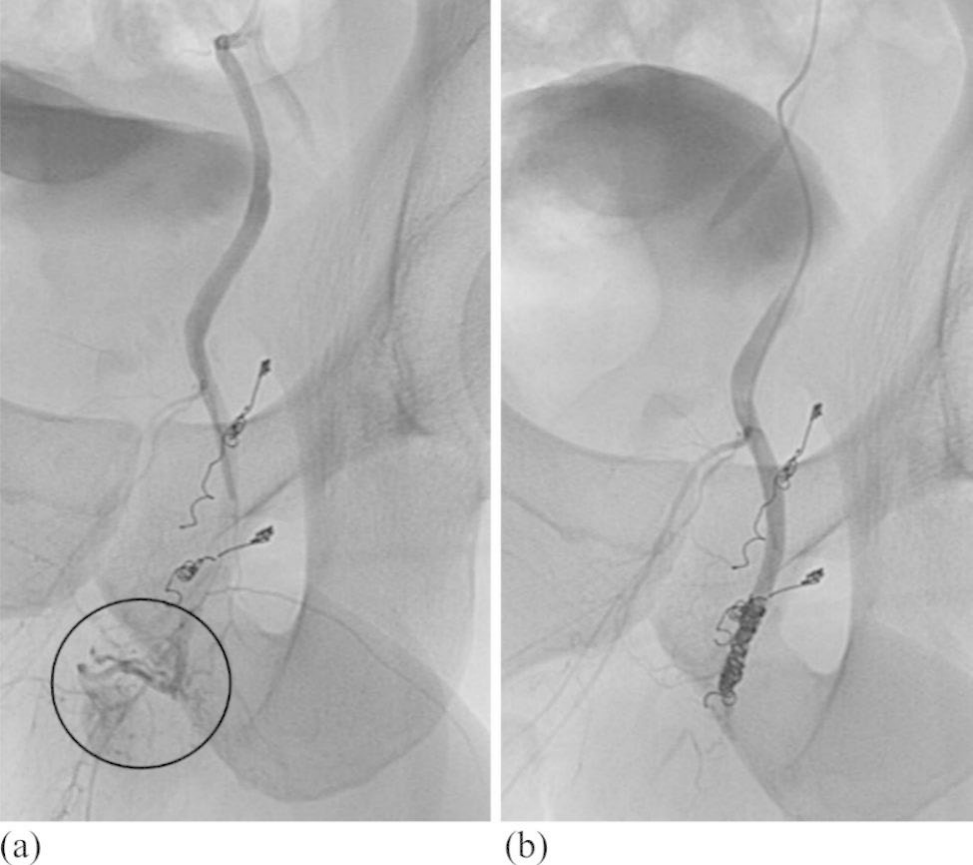
(**a**) Selective angiography of the left pudendal artery revealing an arteriospongious fistula before the second embolization. (**b**) Post-embolization angiographic run showing successful microcoil obliteration of pudendal artery after second intervention

After the first endovascular intervention, the patient reported that erectile function was still inadequate. Three months after the initial procedure, we decided to repeat angiography because the condition was getting worse. An abnormal blush consistent with traumatic arterio-spongious fistula was identified near the previous interventional location (Fig. 2a). Accordingly, we performed another therapeutic embolization with microcoils (Tornado COOK; Interlock Boston), which restored erectile function to normal (Fig. 2b).

**Fig. 3:**
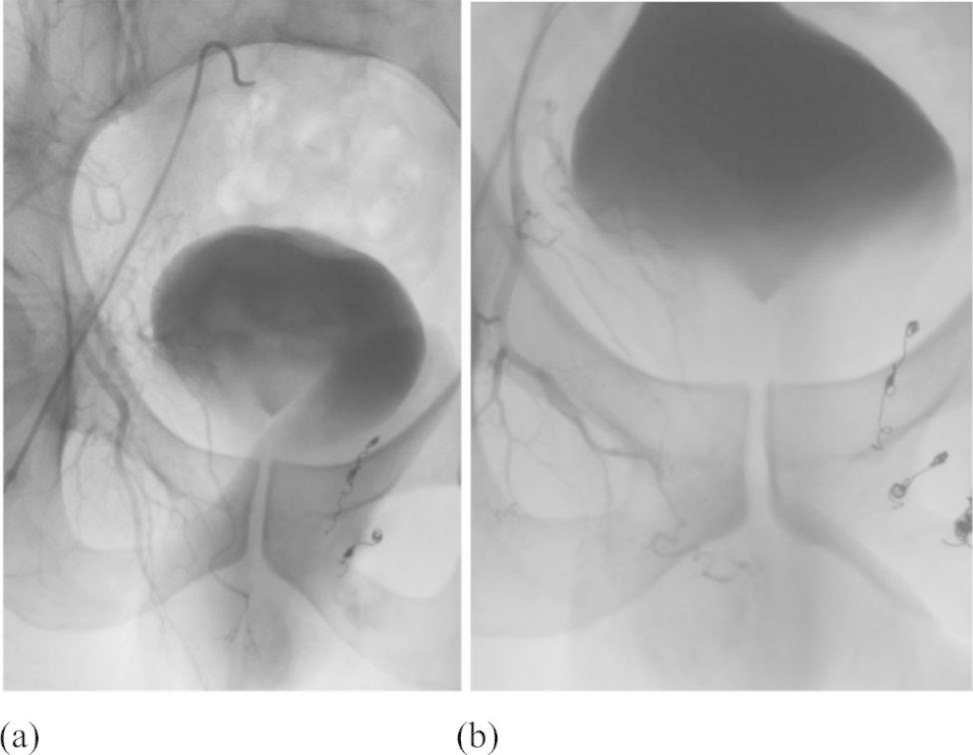
Selective angiography of the right internal iliac artery showed normal anatomy after first (**a**) and second (**b**) embolization on the left side

During the first (Fig. 3a) and second (Fig. 3b) embolization procedures, selective angiography of the right internal iliac artery and its branches was also performed, demonstrating normal anatomy and the preservation of normal vascular patterns.

The interventional radiologist verified the angiographic success of the endovascular treatment by observing the fistula’s exclusion from circulation on the post-embolization control angiogram. The clinical success was assessed by the referring urologist during a final follow-up appointment three months after the second procedure. The urologist confirmed the success of the treatment through a thorough clinical examination and an evaluation of the patient’s satisfaction with erectile function, evaluated using the Sexual Health Inventory for Men (SHIM) questionnaire. Before the embolization therapy, the patient’s erectile dysfunction severity was classified as severe, with a score of 7 points. However, within a few days following the second embolization procedure, the patient reported a complete restoration of fully potent erections, and the patient’s score significantly improved to 23 points. Therefore, no further tests or procedures were required.

## Discussion and conclusions

Clinical evaluation of patients with erectile dysfunction after penile trauma includes a thorough history, physical examination, vascular assessment, and other complementary exams [4]. The most common imaging methods for demonstrating penile vessels are doppler ultrasound (DUS), computed tomography (CT) angiography, and classic digital angiography.

Doppler ultrasonography is the mainstay of diagnosis and follow-up in most cases of arteriovenous fistula described in the literature [7]. DUS confirms the presence of an arteriovenous shunt in the form of decreased resistance in the enlarged afferent artery, a turbulent, high-velocity flow spectrum at the site of the fistula, and a high-velocity and pulsatile flow pattern in dilated, thick-walled draining veins [6]. In our case, due to a high clinical suspicion of post-traumatic vascular damage, the initial DUS assessment was foregone in favour of more invasive diagnostic techniques, such as arteriography, which allowed for potential simultaneous therapeutic intervention.

Contrast-enhanced CT angiography represents the imaging modality of choice when the arteriovenous fistula is related to pelvic trauma. CT angiography shows early filling of a vein adjacent to a contrast opacified artery, indicating a connection between the two vessels [3, 6]. CT angiography or magnetic resonance angiography provides detailed anatomical features of involved vessels and the site and size of arteriovenous fistula and accordingly guides the decision on the best treatment option [6].

Digital catheter angiography is an invasive diagnostic method performed immediately before endovascular treatment to obtain detailed vascular anatomy. It can also be performed diagnostically if non-invasive imaging is insufficient to plan the treatment. Catheter angiography reveals the flow dynamics and precise anatomy of the arteriovenous communication, involved vessels, and collaterals. Furthermore, selective arteriography of the internal iliac artery and superselective angiogram of the internal pudendal artery are usually required to reveal penile arteriovenous fistula [6].

Treatment of traumatic arteriovenous fistula consists of endovascular embolization, surgical ligation, or a combination of both techniques [3]. The main goal of all treatment options is to isolate and close the site of arteriovenous communication. Occlusion or ligation of the proximal feeding artery alone is insufficient because collateral vessels will develop, thus increasing the complexity of the vascular abnormality [6].

Surgical treatment is possible for superficial arteriovenous fistulas with a single afferent artery and few draining vessels. Endovascular embolization is indicated in the treatment of complex arteriovenous fistulas with many feeding and draining vessels. Furthermore, the endovascular approach is also the preferred method for lesions within solid organs or adjacent to critical structures that may be at risk during surgery [6].

Endovascular management involves superselective embolization with coils, gel-foam, or liquid agents. The choice of embolic material depends on the type and size of arteriovenous communication, the flow velocity, and whether the involved vessels can be occluded or require preservation. The most common embolization agent is coil since it allows precise and safe occlusion of arteriovenous fistula [3]. Alternatively, in some cases, embolization can also be performed with gel-foam, polyvinylalcohol (PVA), autologous blood clot, or liquid agents, such as N-butyl cyanoacrylate glue or Onyx [6–8].

Since the first reported endovascular embolization of high-flow priapism using an autologous clot in 1977 by Wear et al., many case reports suggest that embolization offers comparable outcomes using different types of embolic agents [7]. Microcoils enable precise focal occlusion and positioning in the desired vessel. The literature suggests that microcoils are effective in restoring traumatic arteriovenous fistula and are a reliable embolic material even for recurrent cases [7]. Superselective embolization in high-flow priapism is considered a reliable alternative to surgery since it has a success rate of 80% and maintains erectile function in most patients [5].

Arrichiello et al. reviewed 11 published reports from 1990 to 2020 related to high-flow priapism due to arteriocavernosal fistula to analyse the outcome of endovascular treatment. The diagnosis of all patients was based on colour DUS. A total of 117 patients underwent superselective transarterial embolization with an average clinical success rate of 88% (56 to 100%). A recurrence rate of 21% was observed, but in most cases, priapism resolved after a second endovascular treatment; 4 patients underwent surgery. Furthermore, the best outcome regarding embolization material was achieved in 23 patients treated with gel foam, which had the highest clinical success (89%) and the lowest recurrence (13%) rates. The worst clinical success was obtained in 27 patients treated with polyvinylalcohol (70%), with the highest recurrence rate (29%). Patients treated with microcoil embolization had a clinical success rate of 78% and a recurrence rate of 22%, while patients with autologous clot embolization had clinical success and recurrence rates of 72% and 28%, respectively [7].

On the other hand, we found only two cases of endovascular treatment of arteriospongious fistula described in the literature. Glodny et al. in 2007 and Oliveira et al. in 2012 reported cases of traumatic arteriospongious fistulas with erectile dysfunction. The diagnosis of arteriospongious fistula was based on doppler ultrasonography and confirmed by angiography in both cases. Arteriospongious fistulas were successfully treated with coil embolization in both cases without reported recurrence in a follow-up of 6 months [4, 9].

While there are limited reports in the literature describing therapeutic embolization for traumatic arteriospongious fistula with erectile dysfunction, our case demonstrates that coil embolization is a reliable treatment since the symptoms were resolved, and normal erectile function was regained after the intervention.

## Data Availability

All data generated or analysed during this study are included in this published article.

## Declarations

### Authors’ information

***Clinical Institute of Radiology, University Medical Centre Ljubljana, Zaloška cesta 7, SI-1000 Ljubljana, Slovenia.***

Polona Vihtelič, M.D. (polona.vih@gmail.com) & Assoc. Prof. Peter Popovič, M.D., Ph.D. (peter.popovic@kclj.si)

***Department of Urology, University Medical Centre Ljubljana, Zaloška cesta 7, SI-1000 Ljubljana, Slovenia.***

Assist. Simon Hawlina, M.D., FEBU (simon.hawlina@kclj.si).

***Faculty of Medicine, University of Ljubljana, Vrazov trg 2, SI-1000 Ljubljana, Slovenia.***

Assoc. prof. Peter Popovič, M.D., Ph.D. & Assist. Simon Hawlina, M.D., FEBU.

## Abbreviations

***CT***: Computed tomography
***PVA***: Polyvinylalcohol
***DUS***: Doppler ultrasonography
